# MEF2A regulates mGluR-dependent AMPA receptor trafficking independently of Arc/Arg3.1

**DOI:** 10.1038/s41598-018-23440-0

**Published:** 2018-03-27

**Authors:** Ruth E. Carmichael, Kevin A. Wilkinson, Tim J. Craig, Michael C. Ashby, Jeremy M. Henley

**Affiliations:** 1 School of Biochemistry, Centre for Synaptic Plasticity, University of Bristol, BS8 1TD Bristol, United Kingdom; 2 School of Physiology, Pharmacology and Neuroscience, Centre for Synaptic Plasticity, University of Bristol, Bristol, BS8 1TD United Kingdom; 3 Centre for Research in Biosciences, University of the West of England, Bristol, BS16 1QY United Kingdom

## Abstract

Differential trafficking of AMPA receptors (AMPARs) to and from the postsynaptic membrane is a key determinant of the strength of excitatory neurotransmission, and is thought to underlie learning and memory. The transcription factor MEF2 is a negative regulator of memory *in vivo*, in part by regulating trafficking of the AMPAR subunit GluA2, but the molecular mechanisms behind this have not been established. Here we show, via knockdown of endogenous MEF2A in primary neuronal culture, that MEF2A is specifically required for Group I metabotropic glutamate receptor (mGluR)-mediated GluA2 internalisation, but does not regulate AMPAR expression or trafficking under basal conditions. Furthermore, this process occurs independently of changes in expression of Arc/Arg3.1, a previously characterised MEF2 transcriptional target and mediator of mGluR-dependent long-term depression. These data demonstrate a novel MEF2A-dependent mechanism for the regulation of activity-dependent AMPAR trafficking.

## Introduction

Myocyte enhancer factor 2 (MEF2) proteins are a highly conserved family of transcription factors that coordinate protein expression to regulate a wide range of eukaryotic cell functions^1,2^ and are essential for neuronal survival, differentiation and development^3^. Four MEF2 isoforms (MEF2A-D) are expressed in the mammalian brain, which exhibit distinct but overlapping expression patterns^2^. They all act at an A/T-rich MEF2-responsive element (MRE) within the promoter/enhancer region of target genes^4^ to control transcription through the direct recruitment of a range of transcriptional co-factors, including co-activators such as p300/CBP (CREB-binding protein)^5^, and co-repressors such as histone deacetylases^6^.

MEF2 proteins are best characterised as regulators of the structural changes that occur during synapse formation, differentiation and plasticity. More specifically, MEF2 has been proposed to modulate synaptic connections by restricting activity-dependent dendritic spine growth and suppressing formation of orphan presynapses^7–11^. Moreover, MEF2 proteins facilitate robust synapse elimination via a process that involves the Group I metabotropic glutamate receptor (mGluR) mGluR5^12^ and they regulate experience-dependent circuit formation through bidirectional control of excitatory inputs^13^.

AMPA receptors (AMPARs) are a subtype of ionotropic glutamate receptors that mediate the overwhelming majority of fast excitatory neurotransmission in the brain. Developmentally- and activity-regulated changes in the numbers, properties and localisation of AMPARs underpin excitatory synapse formation, stabilization, synaptic plasticity and neural circuit formation^14^. The regulated delivery of AMPARs to and from the postsynaptic membrane, where they contribute to neurotransmission, is a key mechanism underlying the synaptic plasticity required for learning and memory^15^.

Strikingly, it has been reported that MEF2 regulates memory formation *in vivo* through the control of activity-dependent AMPAR trafficking. Learning paradigms in mice decrease MEF2 activity and protein levels in the dentate gyrus and lateral amygdala, which are brain areas required for memory acquisition^16^. Correspondingly, local overexpression of MEF2 decreased, whereas MEF2A/D knockdown increased, performance in memory tasks compared to wild type litter-mates^16^. Memory deficits following MEF2 overexpression were rescued by blocking endocytosis of the AMPAR subunit GluA2, suggesting MEF2 restricts memory formation by promoting internalisation of synaptic AMPARs^16,17^. These data are intriguing because they suggest the direct involvement of MEF2 in memory formation. Importantly, however, these *in vivo* experiments did not address the cell biological mechanisms and signalling pathways underlying the effects of MEF2 on AMPARs.

It has been proposed that the effect of MEF2 on memory is, at least in part, mediated by the activity induced immediate early gene product Arc/Arg3.1 (Arc), which is a key regulator of AMPAR trafficking in certain forms of plasticity^18^. Raised levels of Arc during mGluR-mediated long-term depression (LTD)^19,20^ or synaptic scaling^21^ promote AMPAR internalization via Arc interactions with endophilin-3 and dynamin-2, resulting in decreased AMPAR surface expression^22,23^. Conversely, prolonged inhibition of synaptic activity decreases Arc levels, which leads to reduced endocytosis and increased AMPAR surface expression^21,23^.

Although Arc is a transcriptional target of MEF2^7^, there have been no mechanistic studies explicitly investigating if, and how, manipulation of MEF2 may directly regulate AMPAR surface expression. Moreover, a recent microarray screen has reported that overexpression of a constitutively active form of MEF2A in human neural progenitor cells upregulates transcription of *gria2*, the gene encoding the AMPAR subunit GluA2^24^. Therefore, here we asked if the MEF2A isoform plays a direct role in activity-dependent AMPAR trafficking and the control of synaptic plasticity.

We show that ablating MEF2A expression does not alter total or surface levels of AMPAR subunits under basal conditions. MEF2A is, however, required for GluA2 internalisation following Group I mGluR activation. These findings are the first direct evidence that MEF2A may modulate AMPAR trafficking-dependent memory formation via regulation of the mGluR signalling pathway. Furthermore, contrary to our initial expectations, MEF2A knockdown prevents mGluR-dependent GluA2 internalisation without compromising Arc protein induction, suggesting that this pathway does not involve Arc.

## Results

### Manipulation of MEF2A expression does not affect total AMPAR expression under basal conditions

**Figure 1:**
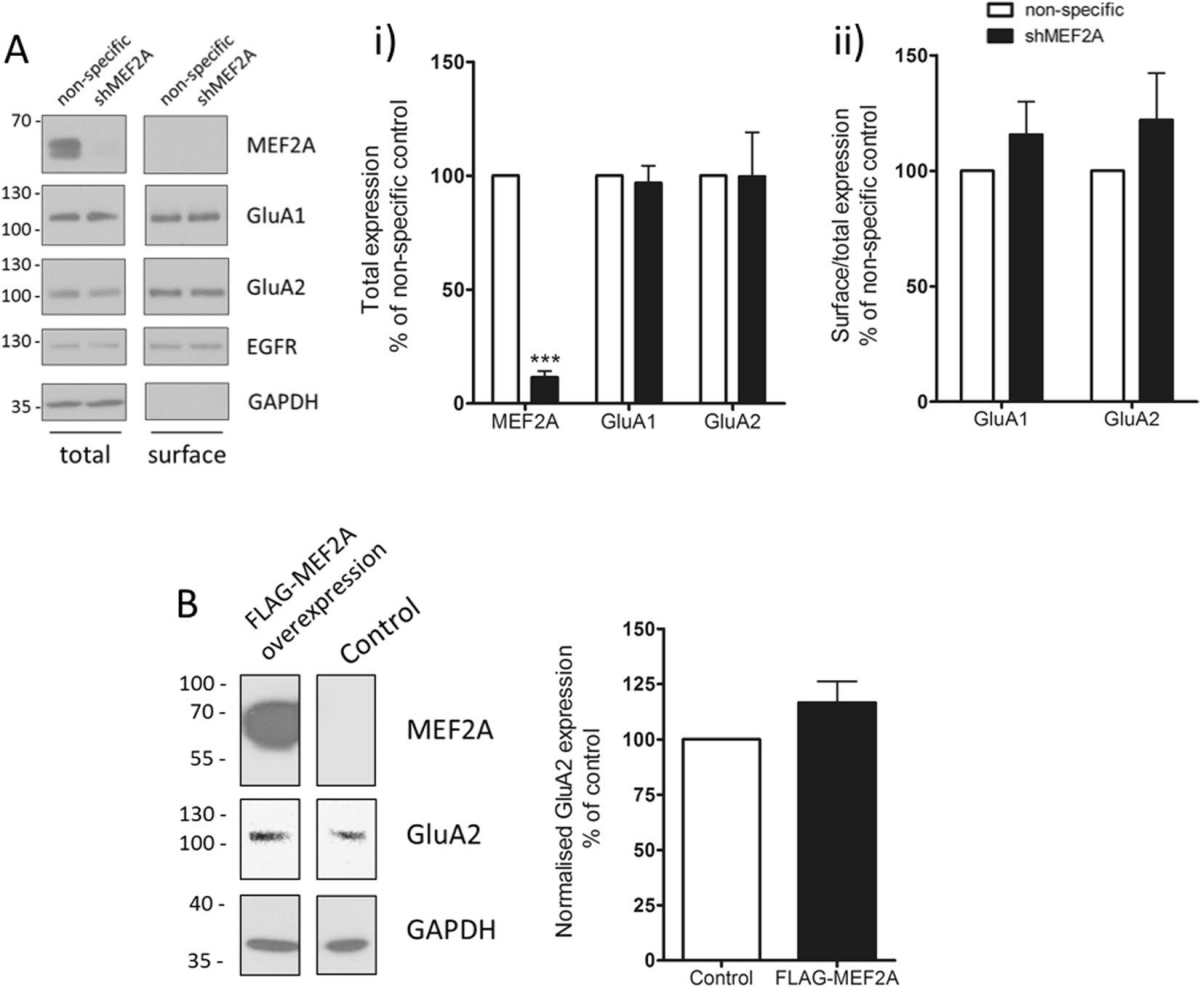
Altering levels of MEF2A does not affect total or surface AMPAR expression under basal conditions. DIV 11 cortical neurons were infected with lentivirus expressing shMEF2A or a non-specific shRNA. Cells were surface biotinylated at DIV 15 to isolate surface proteins, and surface/total protein expression in the cell lysates analysed by Western blotting. (**A**) MEF2A protein expression is significantly reduced in shMEF2A infected cells, but loss of MEF2A does not affect total GluA1 or GluA2 expression (Ai) or the proportion of GluA1 or GluA2 on the surface (Aii) compared to the non-specific control. n = 4, ***p ≤ 0.0001, one-sample t-test. (**B**) DIV 14 cortical neuronal cultures were infected with Sindbis virus encoding FLAG-MEF2A or an empty vector control for 24 h before lysing and Western blotting for total GluA2. Total GluA2 expression was not significantly altered by FLAG-MEF2A overexpression. n = 4.

We first investigated the effects of ablating MEF2A on AMPAR subunit protein expression by infecting rat cortical neuronal cultures with lentiviruses encoding an shRNA sequence targeted to the rat MEF2A transcript (shMEF2A) or a control shRNA sequence. shMEF2A dramatically reduced MEF2A levels five days after infection (Fig. 1Ai). In cortical neurons, loss of MEF2A did not alter total protein levels of the AMPAR subunits GluA1 or GluA2 under unstimulated conditions (Fig. 1Ai). Moreover, Sindbis virus-mediated overexpression of FLAG epitope-tagged MEF2A (FLAG-MEF2A) did not affect basal GluA2 expression (Fig. 1B). Taken together, these data indicate that MEF2A does not modulate AMPAR subunit protein expression under basal conditions.

### MEF2A knockdown does not alter basal levels of AMPAR surface expression

To be functionally active, AMPARs need to be surface expressed at synapses. Thus, although total levels of AMPARs are unchanged it is possible that there could be MEF2A-induced changes in the number of surface expressed AMPARs. Therefore, we tested if MEF2A altered the surface expression of AMPARs under non-stimulated conditions in cultured cortical neurons. Our reasoning was that the inhibitory effect of MEF2 on memory^16,17^ could be attributable to a role in the regulation of constitutive AMPAR surface expression. Our experimental data, however, indicate that this hypothesis is not the case since knockdown of MEF2A had no effect on the proportion of GluA1 or GluA2 expressed on the plasma membrane under basal conditions, as assessed by surface biotinylation (Fig. 1Aii).

**Figure 2:**
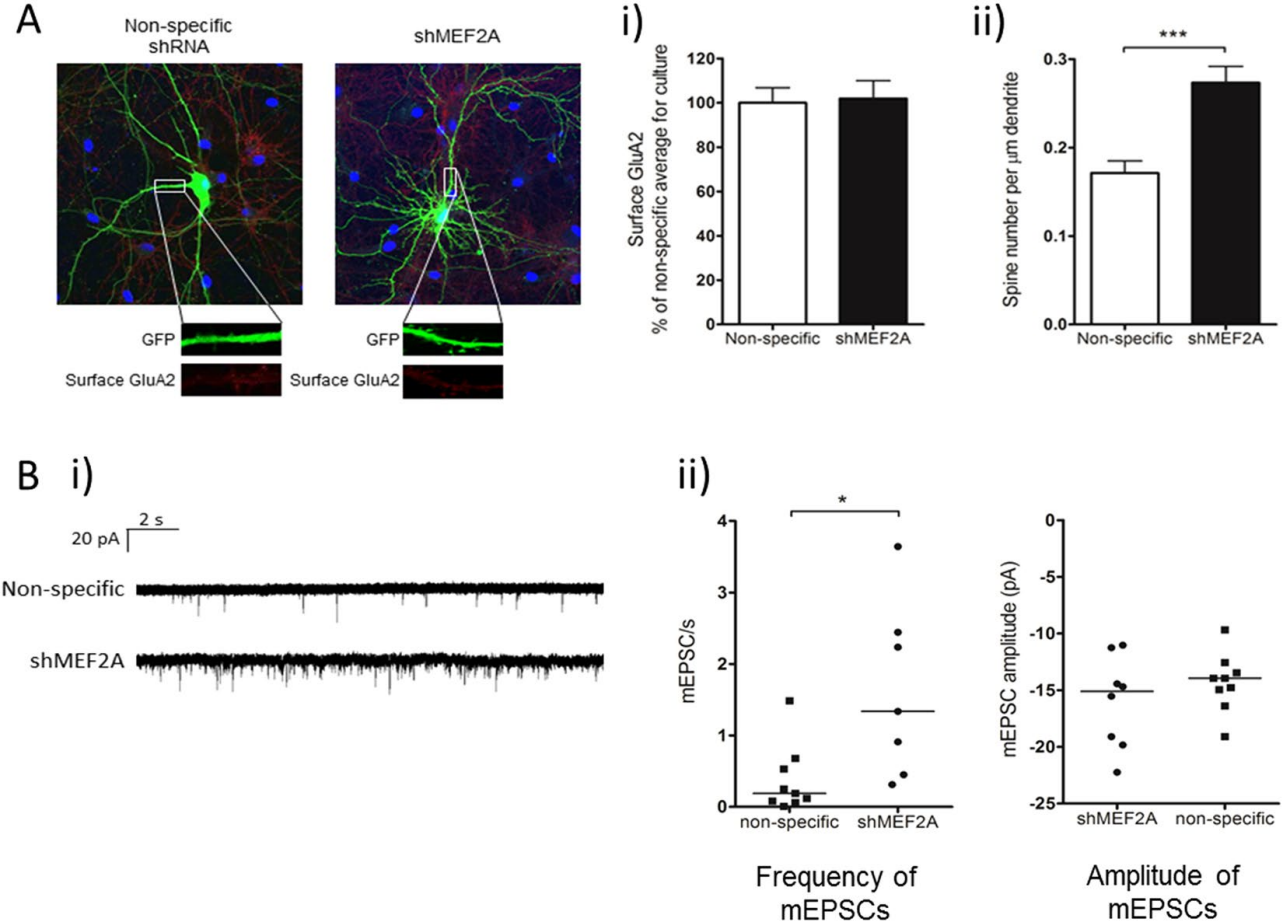
MEF2A knockdown increases synapse number without affecting surface GluA2 levels or synapse strength. DIV 13–15 hippocampal neurons were transfected with shMEF2A, or a non-specific shRNA, coexpressing GFP. (**A**) Surface GluA2 was detected at DIV 18–20 by live immunolabelling, and transfected cells were imaged by fixed confocal fluorescence imaging. Representative images of transfected cells, as identified by GFP expression, showing surface GluA2 staining in primary dendrites. (Ai) Quantification of average surface GluA2 immunofluorescence intensity in primary dendrites showed no significant difference in surface GluA2 expression between shMEF2A and non-specific shRNA expressing cells. Average of three regions of interest per cell, n = 20 cells per condition from 4 independent cultures. (Aii) Dendritic spine density was increased in shMEF2A cells, compared to the non-specific control. Average of three regions of interest per cell, n = 18 (shMEF2A) and 23 (non-specific) from 3 independent cultures, ***p < 0.001, two-tailed unpaired Student’s t-test. (**B**) mEPSCs recorded from DIV 18–20 transfected cells by whole-cell patch clamp at −70 mV. (Bi) Representative mEPSC traces. (Bii) Postsynaptic shMEF2A-expressing cells exhibited a significant increase in mEPSC frequency relative to non-specific shRNA expressing cells (*p = 0.012, two-tailed unpaired Mann-Whitney U test), suggesting an increase in synapse number, whereas there was no significant difference in mEPSC amplitude. n = 7 (shMEF2A) and 9 (non-specific) cells from 5 independent cultures (1–2 cells per culture for each condition).

We next investigated AMPAR surface expression by live cell anti-GluA2 antibody labelling and confocal microscopy in control and shMEF2A transfected rat hippocampal neurons. As shown in Supp Fig. 1, shMEF2A ablated all detectable MEF2A from hippocampal neurons 5 days post-transfection. Levels of GluA2 surface expression were unchanged in the MEF2A knockdown cells, consistent with the surface biotinylation experiments (Fig. 2Ai). However, we observed a significant increase in the density of dendritic spines in cells expressing shMEF2A compared to the control shRNA (Fig. 2Aii), indicative of an increase in synapse number in cells lacking MEF2A.

### Effects of MEF2A knockdown on AMPAR mEPSCs

To define the effect of MEF2A on basal synaptic number and function, we recorded spontaneous AMPAR-mediated postsynaptic currents (mEPSCs) in control and MEF2A knockdown cultured hippocampal neurons. As reported previously for dual MEF2A/D knockdown^7^, we observed a significant increase in mEPSC frequency consistent with an increase in the number of excitatory synapses when MEF2A was knocked down for 5 days (Fig. 2B). There was, however, no difference in the average mEPSC amplitude after ablation of MEF2A, indicating that that the mean number of functional AMPARs at each synapse is unchanged. There was also no difference in mEPSC kinetics between shMEF2A and control cells (Supp Fig. 2), suggesting MEF2A knockdown does not affect basal synaptic AMPAR subunit composition or properties. Together, these results demonstrate that MEF2A does not play a role in the total or surface expression of AMPARs under basal conditions, but is important for maintaining a normal number of functional AMPAR-containing synapses.

Previous studies have reported that knockdown of the A and D isoforms of MEF2 together increases the number of functional excitatory synapses, as determined by dendritic spine density and mEPSC frequency^7^, and reduces activity-induced synapse silencing^25^. However, we found that ablation of the MEF2A isoform alone was sufficient to increase the number of functional AMPAR-containing synapses. Interestingly, this suggests that the four MEF2 isoforms expressed in the brain cannot necessarily compensate for each other, at least in respect to regulation of synapse number, consistent with the different isoforms fulfilling distinct physiological roles. Given that MEF2A was knocked down by transfection in already ‘mature’ cells with morphologically developed spines, the increase in mEPSC frequency observed supports the concept that MEF2A regulates synapse turnover and enhances synaptic silencing^25,26^, rather than just restricting initial synapse formation in development. We attribute our observation that total and surface AMPAR expression do not change, but the number of functional synapses increases in MEF2A knockdown cells, to a redistribution of extrasynaptic surface-expressed AMPARs to synapses in the absence of MEF2A, which could suggest a role in the unsilencing of silent synapses.

### MEF2A knockdown prevents mGluR-induced reduction in AMPAR surface expression

Historically, because of its involvement in synapse development and spine turnover, investigations into the role of MEF2 in plasticity have focussed on structural changes^9,11,27^. However, there is increasing evidence that MEF2 can also regulate learning and memory *in vivo* through changes to synaptic AMPAR expression. For example, MEF2 has recently been implicated in regulating the depression component of *in vivo* ocular dominance plasticity in mice, which is dependent on AMPAR endocytosis^28^. Given its function as a negative regulator of memory *in vivo*, we tested if MEF2A can promote activity-dependent internalisation of GluA2-containing AMPARs, a process that plays a key role in plasticity^29^ and has been proposed to mediate the memory defects associated with MEF2 overexpression^16^.

**Figure 3:**
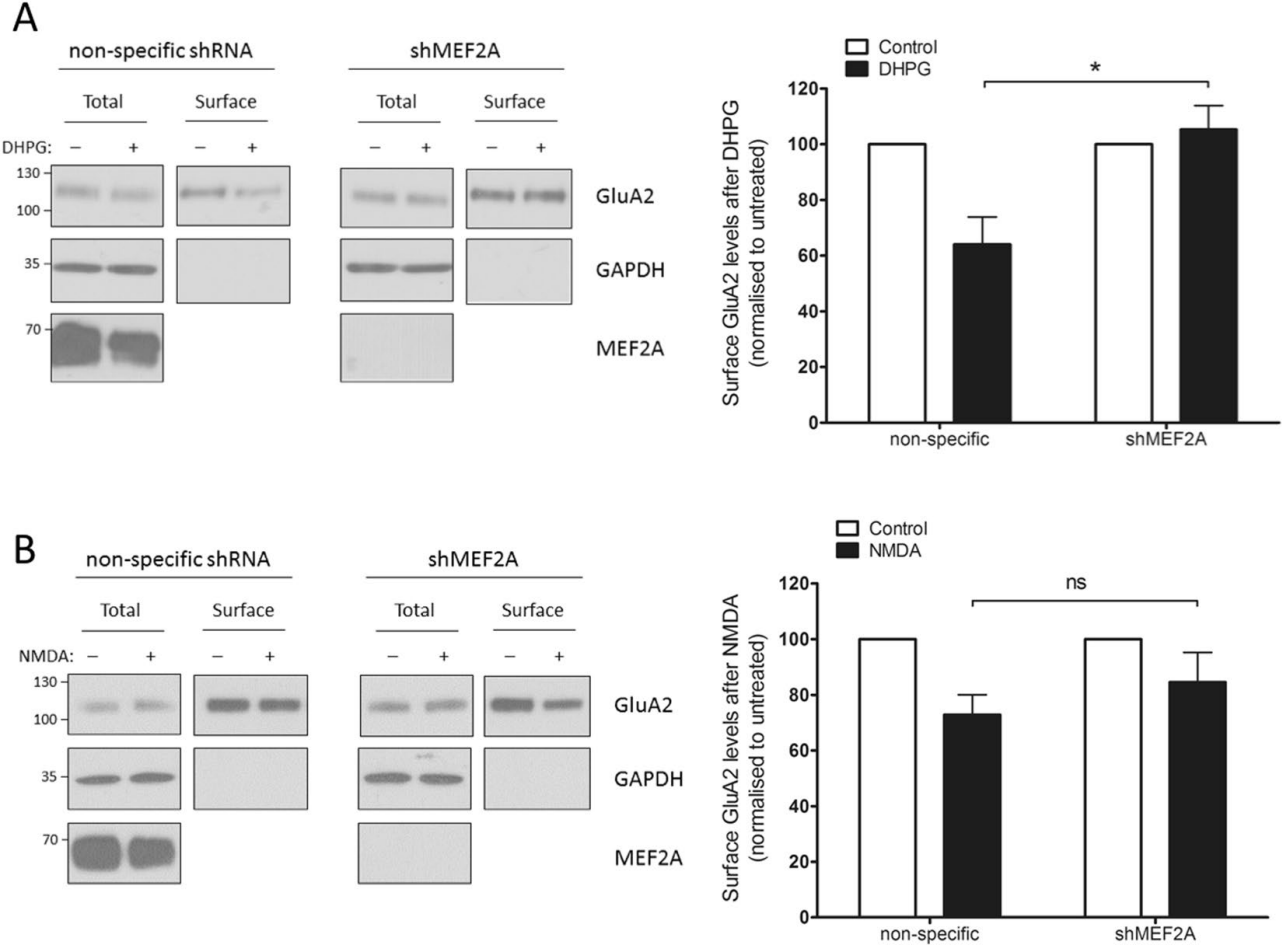
MEF2A knockdown prevents DHPG-induced, but not NMDA-induced, GluA2 internalisation. DIV 11 cortical neurons were infected with lentivirus expressing shMEF2A or a non-specific shRNA control. At DIV 15 cells were treated with the agonist, then incubated in its absence for 10 min to allow for AMPAR trafficking. Cells were surface biotinylated to isolate surface proteins, and surface/total protein expression in the cell lysates analysed by Western blotting. (**A**) Cells treated with 100 µM DHPG for 5 min. Cells expressing shMEF2A expressed a significantly higher proportion of GluA2 on the surface after DHPG treatment (relative to untreated levels) compared to cells expressing the non-specific shRNA control. As before, DHPG caused the proportion of GluA2 on the surface to drop to around 65% of the untreated level in the non-specific shRNA-expressing cells, whereas there was no DHPG-induced decrease in proportional surface GluA2 in shMEF2A-expressing cells. n = 3, *p = 0.0343, two-tailed unpaired Student’s t-test. (**B**) Cells treated with 25 µM NMDA for 3 min. There was no significant difference in proportional surface GluA2 expression after NMDA treatment (relative to untreated levels) between cells expressing shMEF2A versus the non-specific shRNA – in both cases, NMDA caused surface GluA2 expression to drop to ~75% of the untreated levels. n = 5.

Group I mGluR activation evokes AMPAR endocytosis and protein synthesis-dependent long-term depression (mGluR-LTD)^19,30^, at least in part via a pathway that requires Arc^18,20^. In uninfected cultured cortical neurons, the selective Group I mGluR agonist dihydroxyphenylglycine (DHPG; 100 μM, 5 min) caused a significant ~35% decrease in surface expressed GluA2 ten minutes after treatment (Supp Fig. 3). Consistent with the results from uninfected neurons, cells infected with the control shRNA displayed a ~35% decrease in surface GluA2 expression following exposure to DHPG. In stark contrast, however, there was no DHPG-evoked decrease in surface expressed GluA2 in the MEF2A knockdown neurons (Fig. 3A). Furthermore, we confirmed that this effect of MEF2A knockdown on DHPG-dependent AMPAR internalisation was not due to alterations in either the total or surface expression, and thus availability, of the Group I mGluR proteins mGluR1α and mGluR5 (Supp Fig. 4).

In parallel experiments, shMEF2A- and control shRNA-expressing neurons were also stimulated with NMDA, to induce AMPAR internalisation via the NMDAR pathway. In both shMEF2A and control shRNA infected neurons, NMDA evoked a ~25% reduction in surface GluA2 (Fig. 3B), demonstrating that MEF2A is not required for this form of activity-dependent GluA2 internalisation. These data indicate that MEF2A is essential for GluA2 internalisation in response to Group I mGluR activation but does not play a role in NMDAR-evoked GluA2 endocytosis, suggesting MEF2A is specifically required for the Group I mGluR signalling pathway in synaptic plasticity.

We focussed on activity-dependent trafficking of GluA2 because MEF2 has previously been reported to regulate memory *in vivo* through GluA2 endocytosis^16^. We did not explicitly test if GluA1 internalisation is also compromised. However, given that internalisation of both GluA1 and GluA2 is a well-reported consequence of DHPG treatment^19,31^, and the vast majority of synaptic AMPARs consist of GluA1/GluA2 heteromers^32^, we anticipate that DHPG would cause internalisation of both GluA1 and GluA2, and that this would be blocked by MEF2A knockdown.

These results raise the intriguing question of whether MEF2A is regulated by synaptic activity, i.e. if changes in MEF2A expression or function are specifically required for mGluR-dependent AMPAR endocytosis and consequent long-term depression under physiological conditions. Transcriptional effects are likely to occur on a slower time scale than the induction of plasticity, suggesting that MEF2A levels and/or activity could already be ‘primed’ to drive context-appropriate cellular responses. This model would be consistent with the concept of ‘metaplasticity’ in which induction of plasticity processes are themselves plastic, meaning that prior activity generates changes in the subsequent induction of plasticity rather than immediate changes in synaptic transmission^33^. Indeed, MEF2 has been shown to drive functional metaplasticity in *Xenopus laevis* development^34^.

### MEF2A is not required for the induction of Arc protein following Group I mGluR activation

Arc plays a role in the endocytosis of AMPARs required for mGluR-LTD^35^, and *Arc* has been proposed as the MEF2 target gene involved in MEF2-mediated memory impairment *in vivo*^16^. We reasoned that activation of MEF2 may disrupt memory formation and promote mGluR-dependent GluA2 internalisation by increasing levels of Arc to mediate the decreased AMPAR surface expression. We therefore tested the effect of ablating MEF2A on DHPG-induced Arc induction. We detected a significant increase in Arc protein levels 1 hour, but not 10 minutes, after DHPG stimulation, more slowly than in a previous report^20^. While the precise reasons for this remain to be determined, we speculate that one possible reason may be our use of cortical rather than hippocampal cultures.

**Figure 4:**
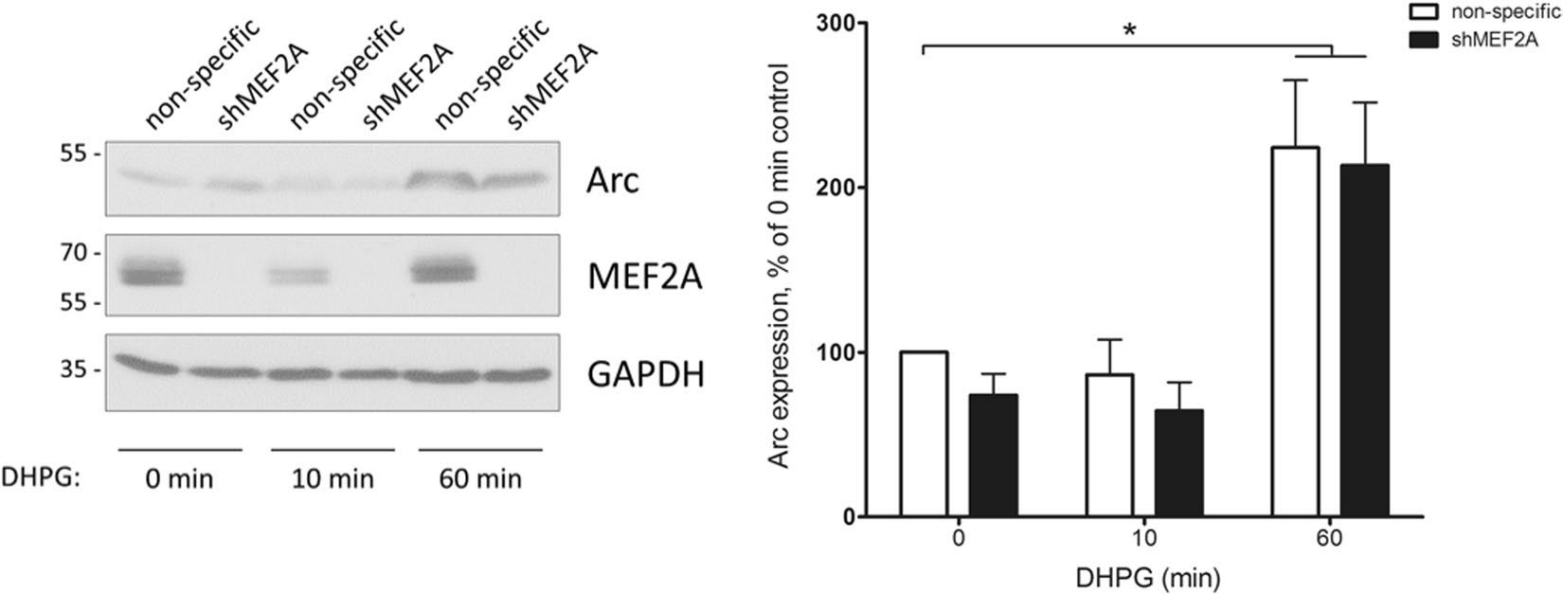
MEF2A knockdown does not prevent the DHPG-induced increase in Arc protein expression. DIV11 cortical neurons were infected with lentivirus expressing shMEF2A or a non-specific shRNA. At DIV 15, cultures were treated with 100 µM DHPG for 0, 10 or 60 min, then lysed and blotted for Arc. For both shMEF2A and non-specific shRNA expressing cultures, 60 min DHPG treatment caused a significant increase in Arc protein levels, relative to untreated cells. However, there was no significant difference in Arc expression between cells expressing shMEF2A versus the non-specific shRNA, with or without DHPG treatment. n = 7, *p = 0.0102 (non-specific) and 0.0111 (shMEF2A), one-sample t-test with Bonferroni correction.

Unexpectedly, MEF2A knockdown for four days in cortical neurons did not reduce Arc protein levels under basal conditions or compromise the induction of Arc following DHPG-mediated Group I mGluR activation (Fig. 4), even though mGluR-dependent AMPAR internalisation was completely blocked in the absence of MEF2A (Fig. 3A). Previous reports have demonstrated compromised Arc induction in response to membrane depolarisation^7^ and brief patterned photostimulation^25^ following MEF2A/D knockdown. It may therefore be the case that Arc expression specifically induced by DHPG is independent of MEF2A activity, or that MEF2A and D can compensate for each other in mediating activity-dependent Arc expression.

Whatever the case, our results suggest that the increase in Arc protein following mGluR stimulation is not sufficient to induce GluA2 internalisation, and an additional MEF2A-regulated factor/pathway is also required. While it remains possible that, although Arc protein induction is maintained in the absence of MEF2A, its mRNA or protein localisation may be altered, this seems an unlikely consequence of loss of a transcription factor. Interestingly, while Arc is necessary for MEF2-dependent spine elimination^12^, it is not sufficient, suggesting that MEF2 may regulate a number of factors that are required for synapse function. Alternatively, since MEF2A is required for Group I mGluR-dependent, but not basal or NMDAR-dependent, AMPAR trafficking, it is possible that MEF2A regulates the transcription of an effector specific to the mGluR signalling pathway. MEF2A could therefore plausibly be necessary for upstream mGluR signalling, rather than controlling the subsequent AMPAR trafficking response directly.

In summary, we have identified a novel requirement for the transcription factor MEF2A in Group I mGluR-dependent AMPAR internalisation, which may represent a molecular mechanism by which it regulates memory formation. How neuronal activity modulates MEF2A to regulate this function, and which MEF2A target genes mediate its effect, are intriguing future questions.

## Methods

### Primary cell culture

Neuronal cultures were prepared from E18 Wistar rats. Briefly, hippocampi and cortex were dissected from E18 brains, dissociated by enzymatic digestion and mechanical dissociation, and then cultured on poly-L-lysine coated glass/plastic in Neurobasal medium supplemented with 5% horse serum, 2% B27, 2 mM Glutamax and 1% penicillin/streptomycin. After 24 h, media was switched for Neurobasal supplemented with 2% B27, 0.8 mM Glutamax and 1% penicillin/streptomycin. Cells were maintained at 37 °C and 5% CO_2_.

### MEF2A knockdown

Short hairpin constructs (shMEF2A: GGGCAGTTATCTCAGGGTTCAA; Non-specific: AATTCTCCGAACGTGTCAC^36^) were cloned under a H1 promoter into a modified pXLG3 vector co-expressing GFP driven by an SFFV promoter^37^.

### Transfection

DIV15 hippocampal neurons were transfected with shRNA-containing pXLG3 constructs using Lipofectamine 2000. 2 μg DNA and 3 μg Lipofectamine 2000 were added to separate tubes containing 100 μl plain Neurobasal media and incubated at RT for 5 min before being combined, vortexed and incubated at RT for 45 min. Hippocampal neurons on glass coverslips were transferred to media lacking penicillin/streptomycin and incubated with the DNA/Lipofectamine mix for 90 min before being returned to their original media.

### Viruses

Lentivirus particles were produced in HEK293T cells (ATCC CRL-3216) maintained in Dulbecco’s Modified Eagle’s Medium supplemented with 10% foetal bovine serum, 2 mM L-glutamine and 1% penicillin/streptomycin. HEK293T cells were co-transfected with the desired pXLG3 vector and second-generation lentivirus packaging plasmids. The HEK293T media containing lentiviral particles was harvested 48 h after transfection. Cells were incubated with lentivirus for 5 days prior to experiments. FLAG-MEF2A was cloned into pSinREP5 for production of Sindbis particles in BHK-21 cells (ATCC CCL-10), which were maintained in Minimum Essential Medium Alpha supplemented with 5% foetal bovine serum and 1% penicillin/streptomycin. Briefly, the pSinREP5 vector and a defective helper vector were linearised and purified then transcribed *in vitro* using mMessage mMachine SP6 transcription kit (Thermo Fisher). RNA was introduced into BHK-21 cells by electroporation using a MicroPulser electroporator. The BHK-21 media containing Sindbis viral particles was harvested 36 h after electroporation. Cells were incubated with Sindbis virus for 24 h prior to experiments.

### Electrophysiology

Transfected hippocampal cultures were immersed in HEPES-buffered saline bath solution (HBS – 137 mM NaCl, 5 mM KCl, 15 mM D-glucose, 25 mM HEPES, 2.5 mM CaCl_2_, 1.5 mM MgSO_4_; pH 7.4) supplemented with 100 nM TTX and 50 μM picrotoxin at room temperature. Transfected cells were identified by GFP expression and recordings from shMEF2A and non-specific shRNA expressing cells were interleaved as far as possible. mEPSCs were recorded by whole-cell patch clamp at a holding voltage of −70 mV using borosilicate glass pipettes with resistances of 4–8 MΩ, filled with voltage clamp solution (135 mM CsMeSO_4_, 8 mM NaCl, 10 mM HEPES, 0.5 mM EGTA, 4 mM Mg-ATP, 0.3 mM Na-GTP, 5 mM QX-314Cl; pH 7.25, 285 mOsM). Recordings from cells with a series resistance less than 25 MΩ and which did not vary by more than 10% during the recording were analysed using Clampfit software (Axon). mEPSCs were identified using template matching and then manually verified. Recordings with fewer than 5 mEPSCs were discarded from further analysis.

### Imaging

For fixed imaging, hippocampal cells on glass coverslips were fixed with 4% PFA for 10 min and permeabilised with 0.1% Triton-X for 10 min. Cells were blocked in 10% horse serum in PBS for 20 min and incubated with primary MEF2A antibody (rabbit monoclonal, 1:250, Abcam ab109420) for 1 h. After washing, cells were incubated with Cy5-conjugated secondary antibody for 1 h then mounted onto slides using Fluoromount-G containing DAPI. For live surface GluA2 labelling, cells were incubated with primary GluA2 antibody (mouse monoclonal, 1:100, Millipore MAB397) for 20 min at 37 °C prior to fixation and incubation with secondary antibody.

Images were acquired using a Leica SP5-II confocal laser scanning microscope attached to a Leica DMI 6000 inverted epifluorescence microscope. Confocal images were captured at 400 Hz with a line average of 4 through a pinhole of one airy unit diameter.

For quantification of MEF2A expression, mean pixel intensity of the MEF2A signal within the nucleus of a transfected cell (nuclear boundary determined by Hoescht staining) was expressed as a percentage of the average mean pixel intensity of the nuclear MEF2A signal of the untransfected cells in the same field of view. For quantification of GluA2 surface expression, the average of the mean pixel intensity of the GluA2 signal from 3 primary dendrites per cell (dendrite boundary determined by GFP expression) was measured.

### Agonist stimulation

Cortical cultured neurons were washed and incubated in isosmotic HBS (137 mM NaCl, 5 mM KCl, 15 mM D-glucose, 25 mM HEPES, 1.5 mM CaCl_2_, 1.5 mM MgSO_4_, pH 7.4) with 0.5 μM tetrodotoxin (TTX) for 15 minutes. Cells were treated with (S)-3,5-DHPG (100 μM, 5 min) or NMDA (25 μM, 3 min) then washed and incubated in HBS with 0.5 μM TTX for a further 10 minutes. For the control conditions, cells were subjected to the same washes and incubations, but the addition of DHPG/NMDA was omitted.

### Surface biotinylation

Cells were incubated for 10 mins with 0.3 mg/ml ice cold solution of Sulfo-NHS-SS-Biotin in PBS, washed three times in PBS and incubated for 2 mins with ice-cold 50 mM ammonium chloride in PBS to quench any excess biotin. Cells were then lysed in ice-cold lysis buffer (150 mM NaCl, 25 mM Tris-HCl pH 7.4, 1% Triton-X 100, with protease inhibitors). Biotinylated proteins were pulled down with streptavidin-agarose beads at 4 °C for 1 h. Proteins were eluted from the beads with 2× Laemmli sample buffer.

### Western blotting

Proteins were separated by SDS-PAGE and transferred to PVDF membrane for Western blotting. Membranes were blocked in 5% w/w non-fat milk powder in PBS-T. Primary antibodies used for blotting were: MEF2A (rabbit monoclonal, 1:5000, Abcam ab109420), GluA1 (rabbit polyclonal, 1:1000, Millipore AB1504), GluA2 (rabbit polyclonal, 1:2000, Synaptic Systems 182103), GAPDH (mouse monoclonal [6C5], 1:10000, Abcam ab8245), Arc (rabbit polyclonal, 1:1000, Synaptic Systems 156003), mGluR1α (rabbit polyclonal, 1:500, Millipore AB1551), mGluR5 (rabbit polyclonal, 1:5000, Upstate Biotechnology 06–451). For protein detection, membranes were incubated with HRP-conjugated secondary antibodies and visualised by enhanced chemiluminescence. Protein bands were quantified by densitometry using ImageJ (NIH). For total protein expression, band intensity of the protein of interest was normalised to the loading control. For proportional surface expression, surface band intensity of the protein of interest was normalised to its band intensity in the total lysate.

### Data availability

All data generated or analysed during this study are included in this published article (and its Supplementary Information files).

## Electronic supplementary material

Supplementary Figures
