# Supplementary material for: MEF2A regulates mGluR-dependent AMPA receptor trafficking independently of Arc/Arg3.1
Source: Sci Rep. 2018 Mar 27;8:5263. doi: 10.1038/s41598-018-23440-0 (PMC5869744; doi:10.1038/s41598-018-23440-0)

MEF2A regulates mGluR-dependent AMPA receptor trafficking independently of Arc/Arg3.1

Ruth E Carmichael[1,3], Kevin A Wilkinson[1], Tim J Craig[3], Michael C Ashby[2] and Jeremy M Henley[1]*

[1]School of Biochemistry and [2]School of Physiology, Pharmacology and Neuroscience, Centre for Synaptic Plasticity, Biomedical Sciences Building, University of Bristol, BS8 1TD, United Kingdom

[3]Centre for Research in Biosciences, University of the West of England, Bristol, BS16 1QY, United Kingdom

Supplementary Information

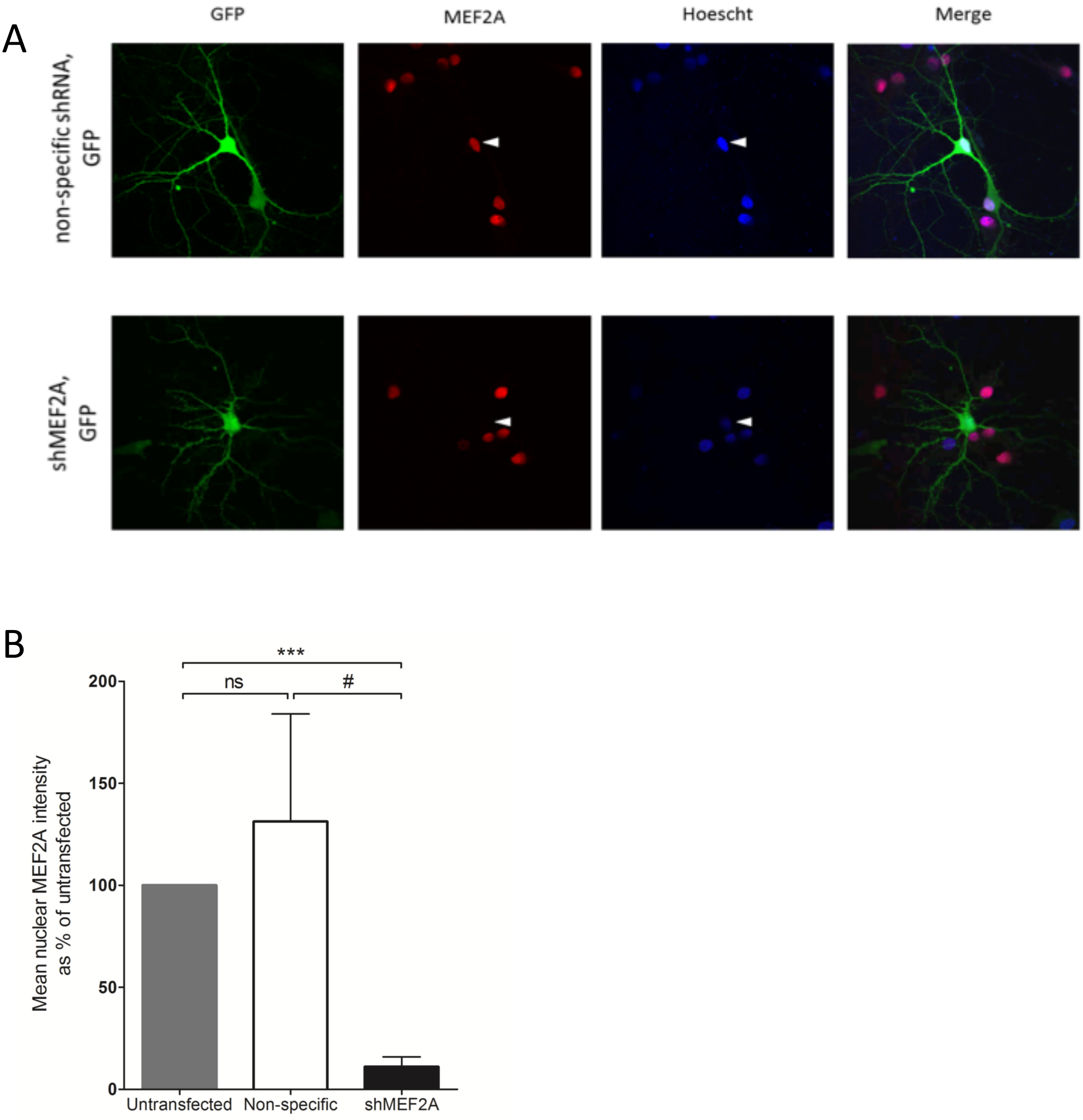

Supplementary Figure 1. shMEF2A transfection knocks down MEF2A expression in cultured hippocampal neurons.

DIV 13-15 hippocampal neuronal cultures transfected with shMEF2A or a non-specific shRNA, co-expressing GFP, immunostained at DIV 18-20 for MEF2A. shMEF2A effectively knocked down endogenous MEF2A (bottom row – no MEF2A immunofluorescence in transfected cell), whereas the non-specific shRNA did not (top row). n = 7 cells (shMEF2A) and 4 cells (non-specific) from three independent cultures. ns = non-significant and *** $p < 0.0001$, one-sample t-test. # $p = 0.0195$, Student's two-tailed unpaired t-test with Welch's correction.

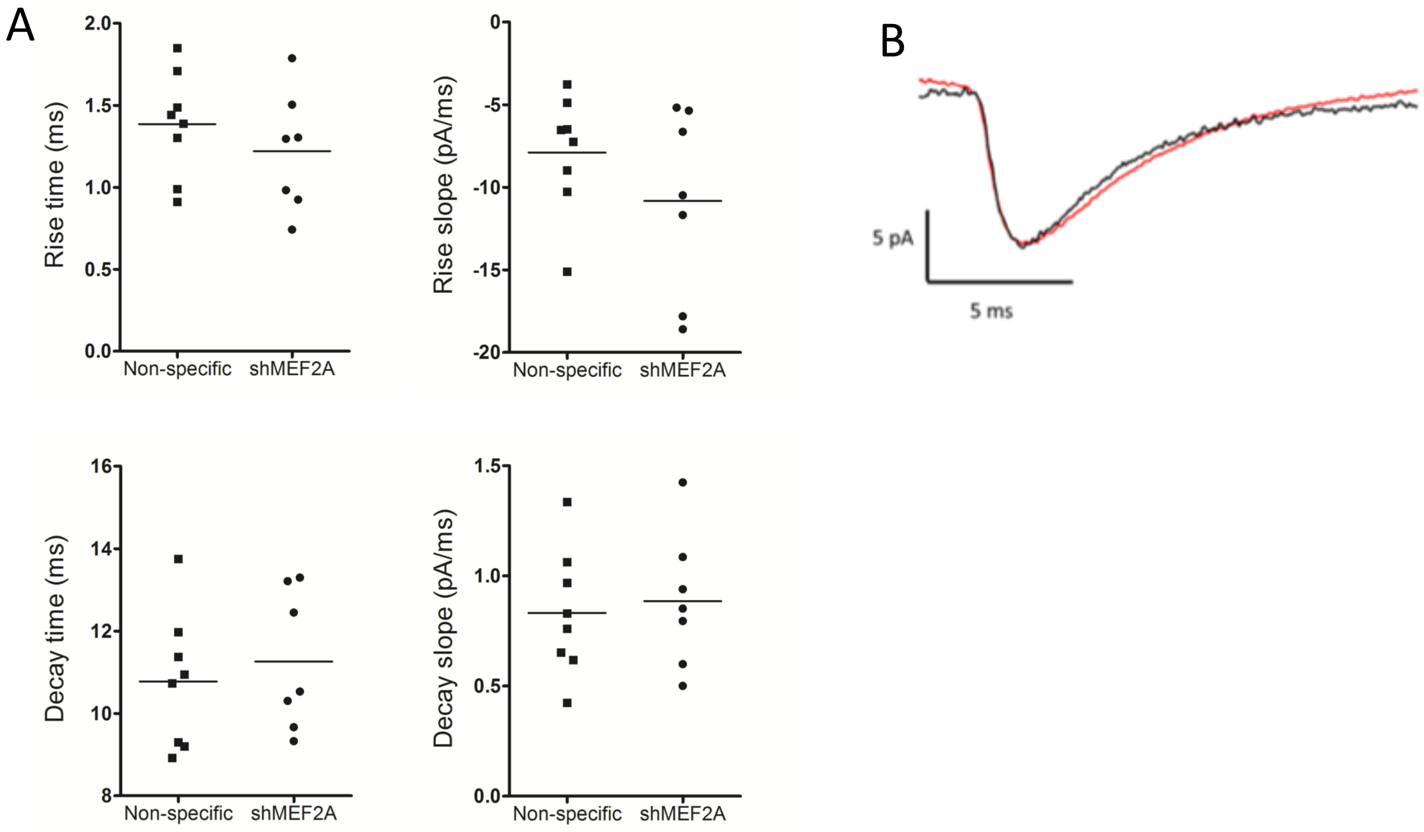

Supplementary Figure 2. **MEF2A knockdown does not affect mEPSC kinetics**

mEPSCs recorded from DIV 18-20 transfected cells by whole-cell patch clamp at -70mV. A) There was no significant difference in the average mEPSC rise time, rise slope, decay time or decay slope (10-90%, average of all recorded events per cell) between shMEF2A and non-specific shRNA expressing cells. n = 7 (shMEF2A) and 8 (non-specific) cells from 5 independent cultures (1-2 cells per culture for each condition). B) Average mEPSC waveform. Red = shMEF2A, black = non-specific shRNA.

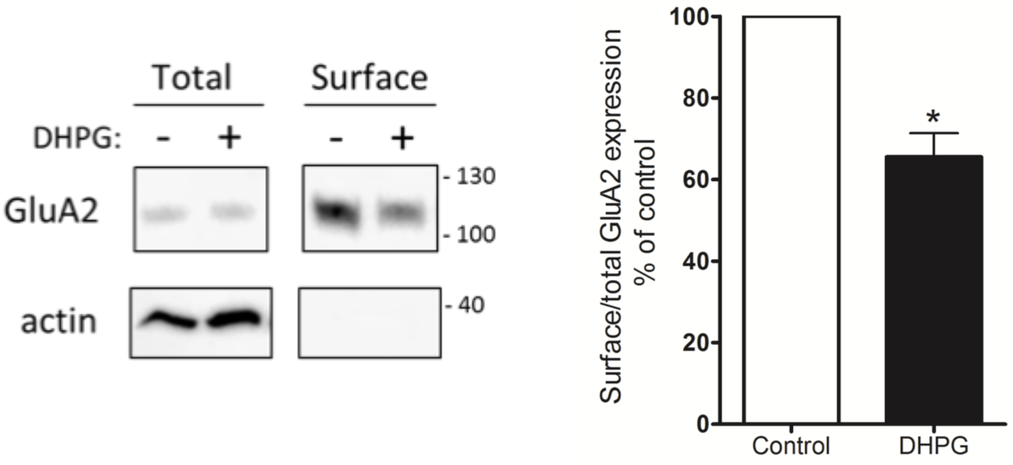

Supplementary Figure 3. DHPG stimulation decreases GluA2 expression on the surface of cortical neurons

DIV 15 cortical neurons treated with 100 μM DHPG for 5 min. After 10 min incubation in the absence of DHPG to allow for AMPAR trafficking, cells were surface biotinylated to isolate surface proteins, and surface/total protein expression in the cell lysates analysed by Western blotting. The proportion of total GluA2 expressed on the surface was reduced by ~35% following DHPG treatment, relative to an untreated control. n = 3, * $p = 0.0274$, one-sample t-test.

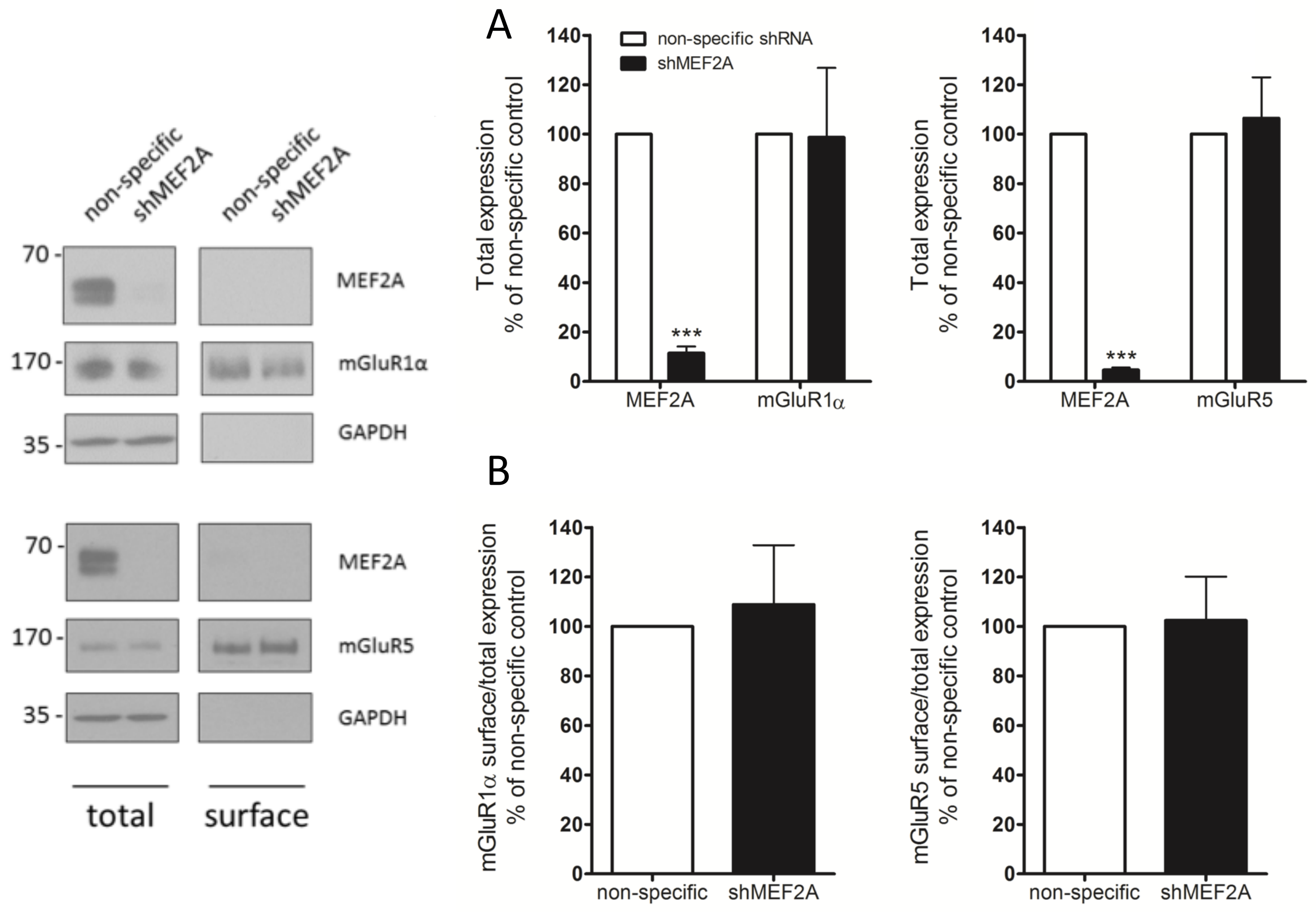

Supplementary Figure 4. MEF2A knockdown does not alter total or surface expression of the Group I mGluR members mGluR1α and mGluR5

DIV 11 cortical neurons infected with lentivirus expressing shMEF2A or a non-specific shRNA. Cells were surface biotinylated at DIV 15 to isolate surface proteins, and surface/total protein expression in the cell lysates analysed by Western blotting. There was no significant difference in either total (A) or surface (B) expression of mGluR1α and mGluR5 between cells expressing shMEF2A or the non-specific shRNA control. n = 4, *** $p \leq 0.0001$, one-sample t-test.

Supplemental Figure 5. Full length, uncropped blots for all figures. Cropped areas are indicated with dashed boxes.

From
Fig 1A)

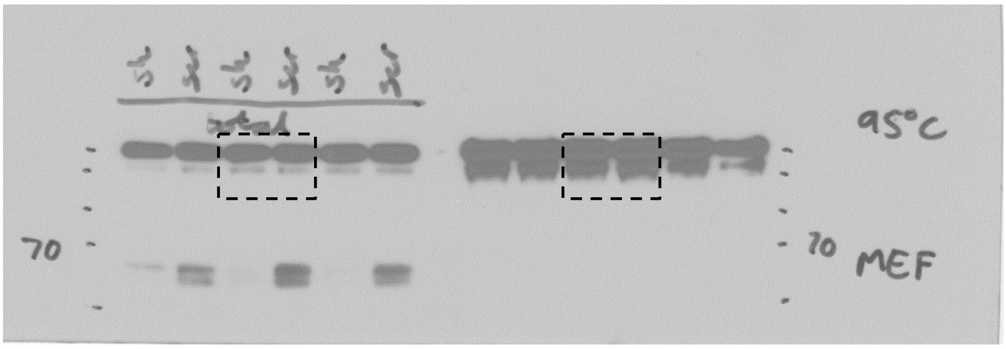

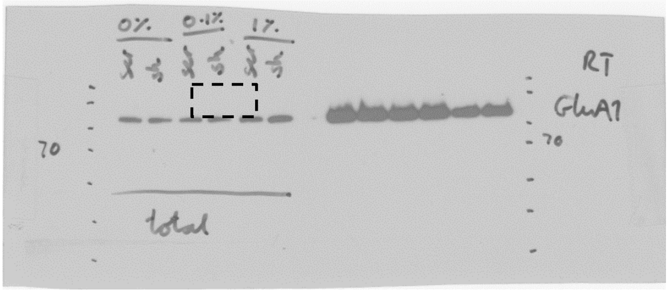

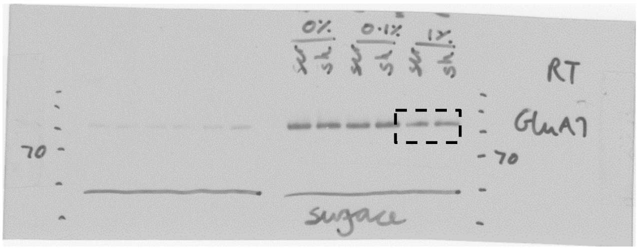

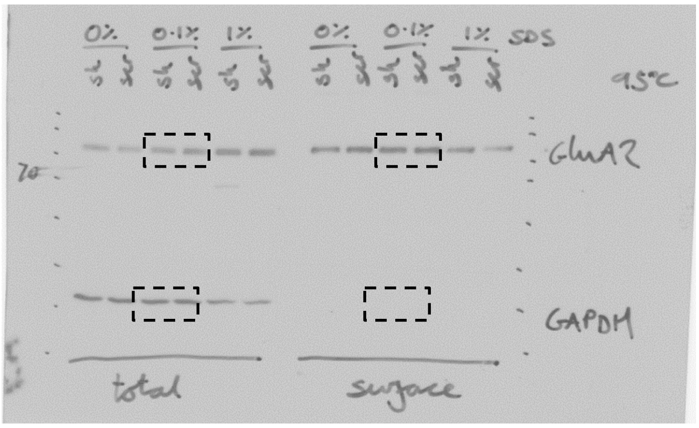

From
Fig 1B)

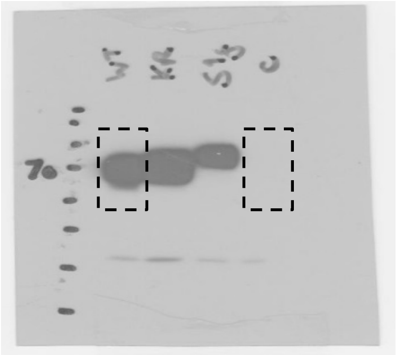

MEF2A

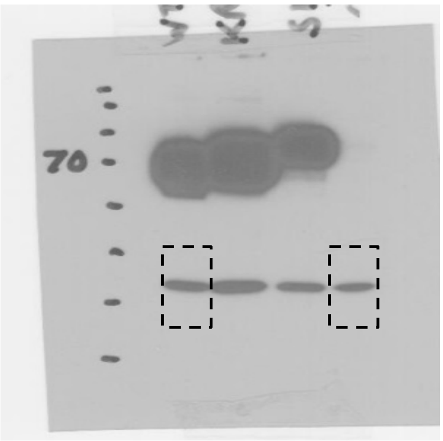

GAPDH

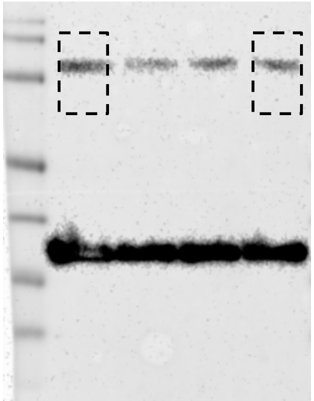

GluA2

Supplementary Figure 5 (continued) – Full length blots

From
Fig 3A)

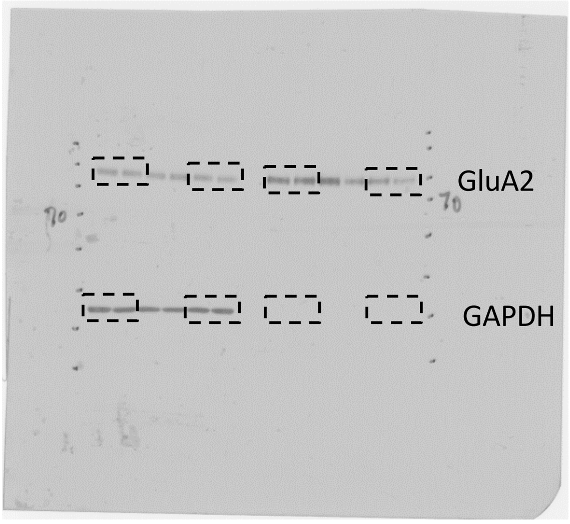

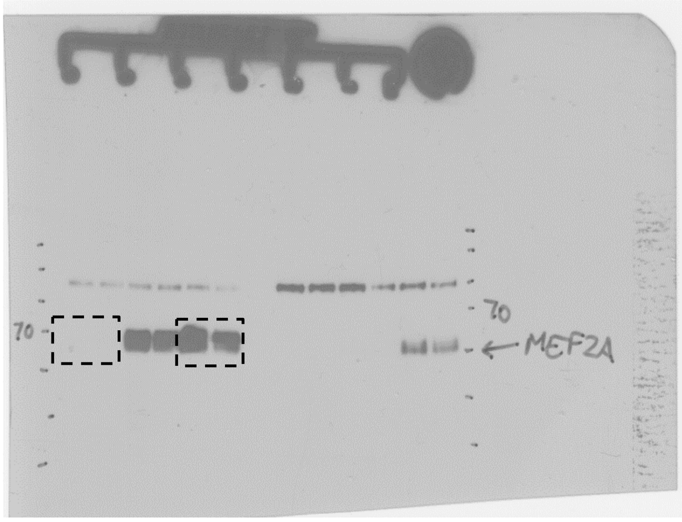

From
Fig 3B)

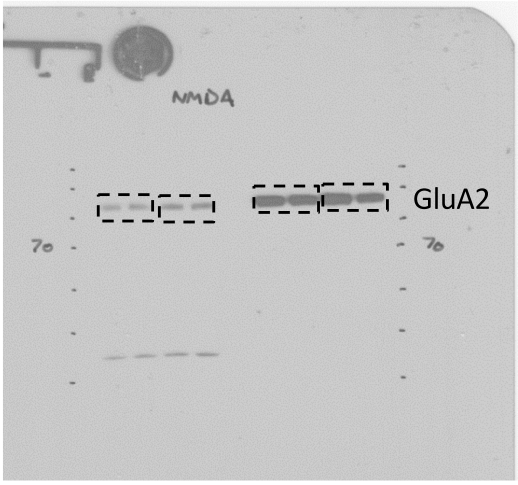

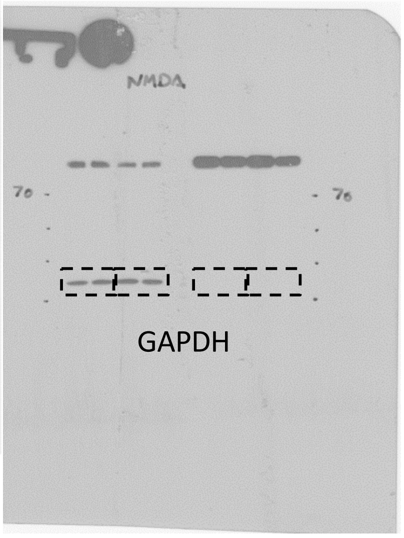

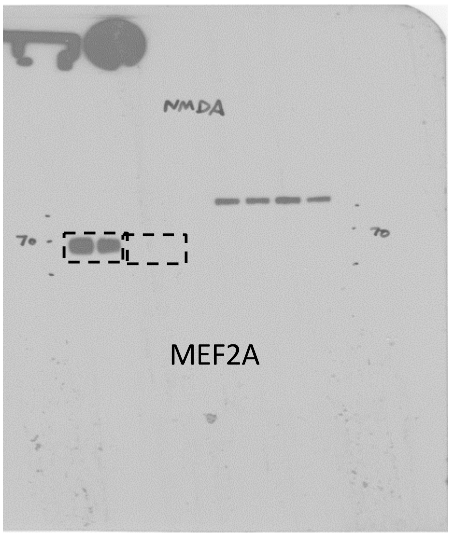

Supplementary Figure 5 (continued) – Full length blots

From Fig 4)

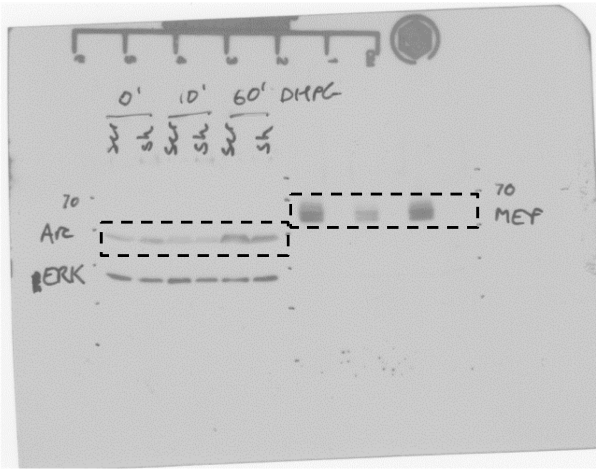

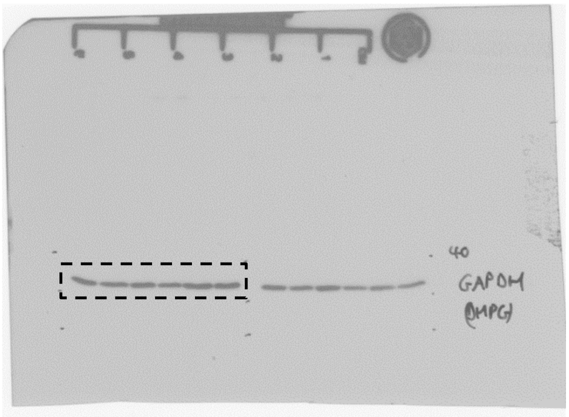

From Supp Fig 3)

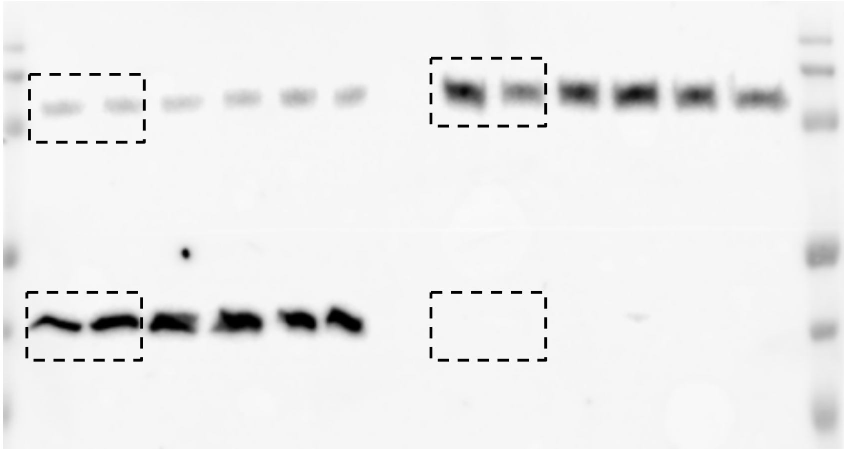

GluA2

actin

From Supp Fig 4)

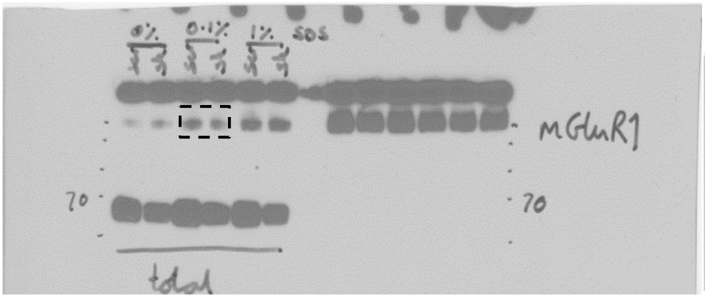

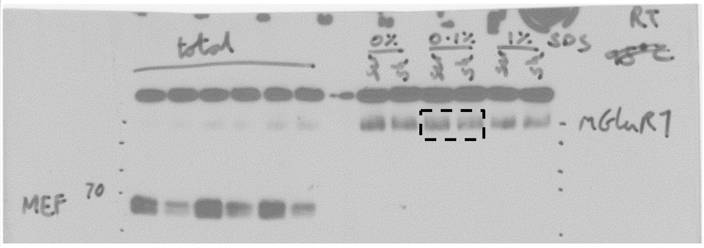

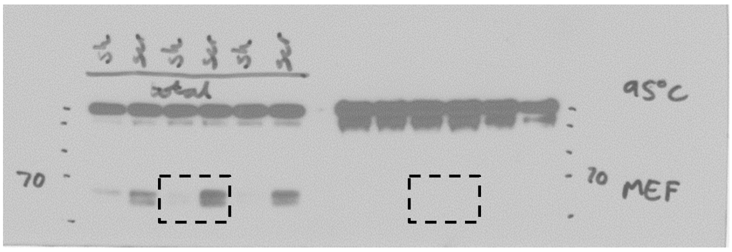

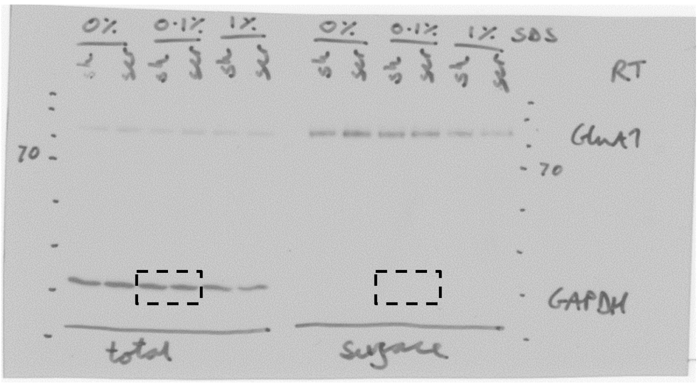

Supplementary Figure 5 (continued) – Full length blots

From Supp Fig 4)

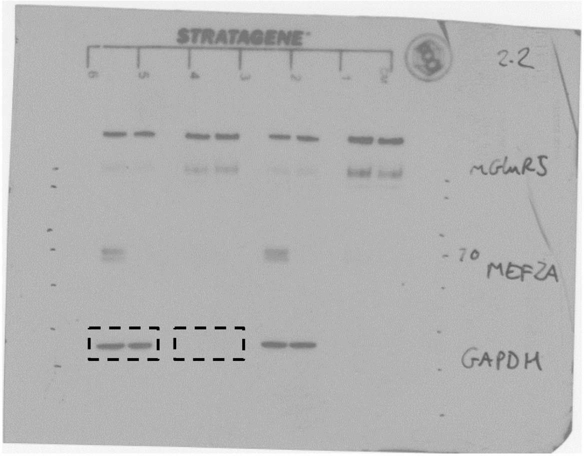

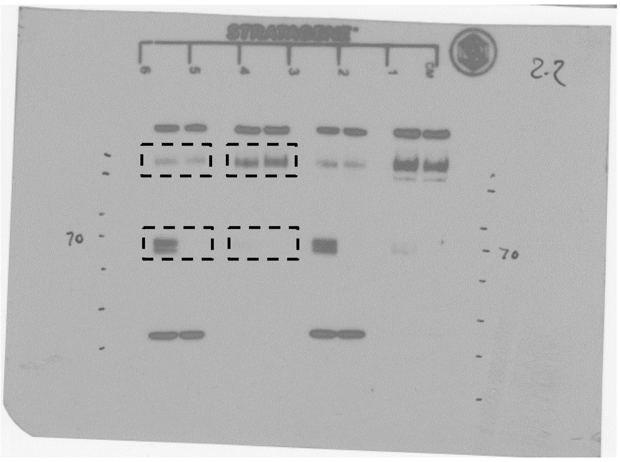

Supplement: Supplementary file 1 — Supplementary Figures [file 41598_2018_23440_MOESM1_ESM.pdf]
